# Antibiotic prescribing practices of medical doctors in a resource-limited setting and the influence of individual perceptions and stewardship support: a survey in three tertiary hospitals in Vietnam

**DOI:** 10.1093/jacamr/dlae064

**Published:** 2024-04-16

**Authors:** Huong Thi Lan Vu, Thuy Thi Thanh Pham, Yen Hai Duong, Quan Anh Truong, Hong Khanh Nguyen, Tu Thi Cam Nguyen, Long Xuan Trinh, Ha Thi Hong Nguyen, Minh Quang Le, Vinh Hai Vu, Duc Minh Chau, Nguyet Thi Huynh, Em Thi Hoang Dung Vo, Hoa Nguyen Minh Le, Thach Ngoc Pham, Todd M Pollack, H Rogier Van Doorn

**Affiliations:** Oxford University Clinical Research Unit, 78 Giai Phong, Hanoi, Vietnam; Partnership for Health Advancement in Vietnam, Beth Israel Deaconess Medical Center, Hanoi, Vietnam; Oxford University Clinical Research Unit, 78 Giai Phong, Hanoi, Vietnam; Oxford University Clinical Research Unit, 78 Giai Phong, Hanoi, Vietnam; Oxford University Clinical Research Unit, 78 Giai Phong, Hanoi, Vietnam; Oxford University Clinical Research Unit, 78 Giai Phong, Hanoi, Vietnam; Vietnam National Children’s Hospital, 18/879 La Thanh, Dong Da, Hanoi, Vietnam; Vietnam National Children’s Hospital, 18/879 La Thanh, Dong Da, Hanoi, Vietnam; Viet Tiep Hospital, 1 Nha Thuong, Cat Dai, Le Chan, Hai Phong, Vietnam; Viet Tiep Hospital, 1 Nha Thuong, Cat Dai, Le Chan, Hai Phong, Vietnam; Dong Thap Hospital, 144 Mai Van Khai, My Tan, Cao Lanh, Dong Thap, Vietnam; Dong Thap Hospital, 144 Mai Van Khai, My Tan, Cao Lanh, Dong Thap, Vietnam; Dong Thap Hospital, 144 Mai Van Khai, My Tan, Cao Lanh, Dong Thap, Vietnam; National Hospital for Tropical Diseases, 78 Giai Phong, Hanoi, Vietnam; National Hospital for Tropical Diseases, 78 Giai Phong, Hanoi, Vietnam; Partnership for Health Advancement in Vietnam, Beth Israel Deaconess Medical Center, Hanoi, Vietnam; Oxford University Clinical Research Unit, 78 Giai Phong, Hanoi, Vietnam; Centre for Tropical Medicine and Global Health, Nuffield Department of Medicine, University of Oxford, Oxford, UK

## Abstract

**Objectives:**

To understand antibiotic prescribing and influencing factors to inform antimicrobial stewardship (AMS) interventions to reduce unwanted consequences of antibiotic use in hospitals in Vietnam, a lower-middle-income country in Asia.

**Methods:**

We conducted a cross-sectional study of doctors at three tertiary hospitals using non-probability convenience sampling, through a paper-based (Hospitals 1 and 2) or electronic (Hospital 3) survey. Questions included items on perceptions regarding antibiotic resistance and AMS, prescribing practices, knowledge, demographics and training. We used principal components analysis and mixed-effects models to examine practices and identify influencing factors.

**Results:**

Among 314 surveyed participants, 61%, 57% and 59% in Hospitals 1, 2 and 3, respectively, felt certain about the appropriateness of their antibiotic prescriptions. In total, 9% reported sometimes prescribing antibiotics when not needed to meet patients’ expectations, and 13% reported doing so to avoid perceived complications. Higher prescribing confidence was found among those with positive perceptions about AMS (*P* < 0.0001), whereas negative perceptions about colleagues’ practices reduced this confidence (*P* < 0.0001). Individual preference for branded antibiotics was associated with more unnecessary prescribing whereas having higher prescribing confidence decreased the habits of prescribing when not needed.

**Conclusions:**

This study provides important implications for design of hospital interventions to address influencing factors on antibiotic prescribing in Vietnam and similar resource-limited settings. Specific interventions should target improving knowledge through education and training for doctors, enhancing the support from the AMS team, and promoting guidelines and policies for appropriate antibiotic use in hospital.

## Introduction

Doctors’ antibiotic prescribing decisions are influenced by many factors, including patients’ expectation and doctors’ fear of possible complications and losing patients.^1^ Additionally, doctors’ knowledge about antibiotics and awareness about antimicrobial resistance (AMR) also play an important role in appropriate prescribing practices, mediated by years of clinical experience and social and psychological factors.^2^ A cross-sectional study in China showed that doctors with more knowledge of antibiotics prescribed less antibiotics and antibiotic combinations.^3^ A worldwide survey among surgeons emphasized the importance of providing periodic reports of local AMR patterns and communication with microbiologists to support them in antibiotic prescribing.^4^ A recent review showed that doctors in resource-limited hospitals generally had good levels of theoretical knowledge and confidence in prescribing antibiotics; however, many of them were unaware of the local resistance patterns.^5^ This raised a concern for irrational antibiotic use, treatment failures, and increased resistance in low- and middle-income countries (LMIC).

Antimicrobial stewardship (AMS) programmes have been increasingly implemented worldwide to optimize antibiotic prescriptions in hospitals, while preserving them for future use.^6^ In the context of AMS, doctors need to go through a complex decision-making process governed by AMS regulations and restrictions and psychological barriers such as personal beliefs and fears.^7^ Disease severity, uncertainty about the source of infection, and personal acceptability thresholds for coverage of empiric treatment were shown to increase the selection of combination therapies and broad-spectrum antibiotics among doctors in Vietnam.^8^ Previous reviews have shown that AMS implementation in LMIC is likely to be effective but requires interventions to be tailored to the local contexts.^9–11^ Studies in these settings have also raised a concern about low levels of AMS-related knowledge among hospital staff, including doctors, which necessitates the contextualized educational interventions and capacity-building activities.^12,13^ In addition, a number of external factors such as promotional activities from pharmaceutical companies and drug supply (costs, availability and quality) have been shown to affect the prescribing practices and impact of AMS programmes.^14–16^

Vietnam is a lower-middle-income country in Southeast Asia with rising burdens of AMR in a number of important bacterial pathogens, as shown in the surveillance data.^17,18^ The country developed its first National Action Plan on AMR in 2013, and recently released the National Strategy on AMR control for 2023–2030, vision to 2045. AMS implementation is among the main strategies identified, with the national guidelines on hospital-based AMS implementation first issued in 2016 and updated in 2020. In addition, training opportunities on AMR for healthcare staff, including doctors and pharmacists, have increased in recent years but are limited to the national and provincial levels.^19^ There is a high level of antibiotic use in hospitals; a study reported varying rates of antibiotic use across clinical departments at provincial level, ranging from 500 to 1000 days of antibiotic therapy per 1000 patient days.^20^ Studies also showed some initial impact of AMS implementation in hospitals across the country^20–22^ despite numerous challenges remaining.^16^ Surveys of knowledge, attitudes and practices (KAP) have been used to understand the current gaps in antibiotic treatment practices to help with design of targeted interventions.^20^ Here we analysed pooled data from three KAP surveys among doctors in tertiary hospitals in Vietnam to understand antibiotic prescribing and influencing factors within the hospital context to inform stewardship interventions for better patient care and reduce unwanted consequences of antibiotic use.

## Methods

### Study settings

We conducted these KAP surveys in two provincial hospitals (Hospital 1: 2000-bed capacity, 27 wards; Hospital 2: 1000-bed capacity, 26 wards) in February–March 2020 and a national paediatric hospital (Hospital 3: 1743-bed capacity, 25 wards) in January–March 2018.

At each hospital, an AMS committee had been established following the national guidelines.^23,24^ An antibiotic restriction policy was applied at all hospitals for a limited number of antibiotics as per the guidelines. Expertise for infection management was available at all three hospitals including infectious disease specialist, clinical pharmacist and clinical microbiologist and could be accessed through direct communication and hospital-wide consultations on difficult cases.

### Study design

This survey was conducted as a preintervention assessment in three hospitals. Study samples were selected using convenience sampling of clinical wards that was specific to each hospital based on the feasibility of data collection. At each hospital, the director board made the decision on the list of clinical wards to participate in the survey. The survey form was then distributed to all doctors of the selected wards for completion either in paper-based or electronic form. Completion and return of the form indicated consent to participate in the study. At Hospital 1, paper forms were distributed to all doctors of 8/27 clinical wards [sampling fraction for doctors = 61/420 (14.2%), response rate = 61/61 (100%)]. The eight study wards at Hospital 1 included two ICUs (one surgical and one internal), two surgical wards (traumatology, gastroenterology) and four internal wards (one infectious diseases, two general medicine and one oncology). At Hospital 2, paper forms were distributed to all doctors available during the survey time period at all 26/26 clinical wards [*n* = 120, sampling fraction: 120/227 (52.9%), response rate: 120/120 (100%)]. At Hospital 3, an electronic survey link was sent to all doctors of 25/25 wards [sampling fraction: 314/314 (100%), response rate: 172/314 (54.8%)].

The purposes of the survey were to establish the baseline knowledge, misconceptions, attitudes, beliefs and behaviours of doctors in antibiotic prescribing to help identify gaps and issues that could be targeted in the AMS programmes of the study hospitals. Therefore, we used local guidelines on antibiotic use and AMS issued by Vietnam’s Ministry of Health in combination with international guidelines to formulate the questions. For knowledge of antibiotic treatment, we developed 16 questions based on the national antibiotic treatment guidelines.^25^ We developed 9 questions about perceptions and attitudes of antibiotic use and resistance, 7 about perceptions and attitudes about AMS, and 12 about prescribing practices based on national and international guidelines and toolkits related to antimicrobial resistance, infection control and antimicrobial stewardship (see Supplementary Method; available as Supplementary data at *JAC-AMR* Online). We selected the questions deemed relevant for the practice of clinical care for infectious diseases in Vietnam’s hospitals. We also asked for information about participants’ gender, age, clinical specialty, years of clinical experience, current job position, and training on antibiotics in the past year.

### Data analysis

Data were summarized in absolute and relative frequencies and analysed using R program.^26^ Knowledge questions were scored (1 for the correct answer, 0 for incorrect answers) and the sum of the scores was calculated for each participant. We reported data for all three hospitals in total and for each hospital if relevant, with the corresponding denominators. Because there were missing data (participants did not respond to a specific question or statement), we reported the denominators to show the actual total number of participants responding to specific questions and statements.

Principal components analysis (psych package)^27^ was used to examine the components underlying the statements related to perceptions, attitudes and practices. One component can be considered as a high-level variable that describes a combination of interrelated statements extracted from the original set of statements in the survey. Through this process, we could summarize the results of responses to all statements of the survey in a small number of variables. In this analysis, a total of six components were extracted based on two criteria: (i) the eigenvalues of the real data were less than the corresponding eigenvalues of a random data set of the same size in a parallel analysis (fa.parallel function),^28^ and (ii) the components identified were interpretable. The eigenvalue represents the total amount of variance that can be explained by a given component and is useful in helping to determine the optimal number of components that we should extract during the principal components analysis. A composite score for each of the six identified components was then calculated and these components were then used as variables in the multivariable linear mixed effects models to examine associations with antibiotic prescribing practices. These multivariable linear mixed effects models (lme4 package)^29^ aimed to examine the fixed effects of variables on antibiotic prescribing practices. We included all variables in the same model for each outcome, with hospital included as a random effect.

### Ethics

The study was approved by the Oxford Tropical Research Ethics Committee (OxTREC Reference: 526-19, 15 May 2019) and Institutional Review Boards at the National Hospital for Tropical Diseases (No.8-HDDD/NDTU, 31 May 2019), Viet Tiep Hospital (4767-QD/BVVT, 23 May 2019) and Dong Thap Provincial Hospital (Approval dated 16 May 2019). The study was also approved by Vietnam National Children’s Hospital (Approval dated 12 January 2018) and categorized as exempt from ethics review by the Institutional Review Board at the Beth Israel Deaconess Medical Center.

## Results

### Participant characteristics and their knowledge of antibiotic prescribing

In total, 319 doctors from three hospitals completed the survey (Table 1). Clinical specializations varied greatly among surgery, ICU, internal medicine and infectious disease across the three hospitals. Most participants (93.3%, 97.5% and 69.2%, respectively) had received antibiotic training in the past year; however, nearly half of participants stated the inadequacy of the training (44.3%, 24.2% and 44.9%, respectively). Of all participants, the mean knowledge score was 9.77 out of 16 (95% CI: 9.47–10.01), with a higher mean score for Hospital 3 (10.43) compared with the mean scores for Hospital 1 (8.64) and Hospital 2 (9.13). The proportions of participants with correct answers for each of the 16 knowledge questions are presented in Table S1.

**Table 1.** Summary information on demographics, training and knowledge of medical doctors participating in the KAP survey at three hospitals^a^

Variable	Hospital 1 (*n* = 61)	Hospital 2 (*n* = 120)	Hospital 3 (*n* = 138)
Male sex	45 (73.8%)	81 (67.5%)	36 (26.1%)
Age, mean, y (SD)	36.3 (9.7)	36.6 (11.3)	35.9 (6.4)
Specialty			
ICU	19 (31.1%)	18 (15.0%)	29 (21.0%)
Internal medicine	9 (14.8%)	59 (49.2%)	44 (31.9%)
Infectious diseases	6 (9.8%)	9 (7.5%)	12 (8.7%)
Surgery	27 (44.3%)	34 (28.3%)	12 (8.7%)
Others	—	—	41 (29.7%)
Years of clinical experience at current hospital			
<1 year	10 (16.4%)	22 (18.3%)	6 (4.3%)
1–5 years	18 (29.5%)	35 (29.2%)	45 (32.6%)
6–10 years	9 (14.8%)	16 (13.3%)	55 (39.9%)
>10 years	24 (39.3%)	47 (39.2%)	32 (23.2%)
Managerial position	16 (26.2%)	30 (25.0%)	11 (8.0%)
Training on antibiotics in past year (yes)	56/60 (93.3%)	116/119 (97.5%)	90/130 (69.2%)
Perceived training was inadequate^b^	27/61 (44.3%)	29/120 (24.2%)	62/138 (44.9%)
Antibiotic treatment knowledge score^c^	8.64 (2.70)	9.13 (1.99)	10.43 (2.29)

^a^Thirty-four participants were excluded from the analysis due to missing data confirming they were prescribers, resulting in a total of 319 participants included in the analysis.

^b^Agreed that training on antibiotic prescribing and use at the hospital was inadequate.

^c^Antibiotic treatment knowledge was scored out of 16 (1 for each correct answer to 16 questions on antibiotic prescribing treatment practices), data presented are mean (SD).

Questions with low proportions of participants providing correct answers across three hospitals mainly concern the adjustment of antibiotic choices based on blood culture results (31.7%, 8.1% and 54.5% in Hospitals 1, 2 and 3, respectively), identifying the antibiotic that does not require dose adjustment in case of impaired renal function (45.0%, 66.4% and 35.6%), choice of level determinations in monitoring vancomycin therapy (24.6%, 19.2% and 56.8%), amikacin dosing in a patient with impaired renal function (34.4%, 73.8% and 41.7%), antibiotic choice in abscess management (24.6%, 39.2% and 61.4%), choice of antibiotics used in appendectomy (45.9%, 55,8% and 62.1%), antibiotic decision in asymptomatic bacteriuria (23.3%, 41.0% and 54.5%), antibiotic therapy for community-acquired pneumonia (59.0%, 20.0% and 65.9%) and hospital-acquired pneumonia (66.1%, 59.2%, and34.8%), and antibiotic choice for a patient with a history of anaphylaxis to penicillin (48.3%, 33.9% and 50.8%). These present the key gaps in the knowledge of doctors in the three hospitals that need to be addressed in the ongoing training and educational activities of the AMS programmes.

### Antibiotic prescribing practices and perceptions about AMR and AMS

Of all participants, 61%, 57% and 59% in Hospitals 1, 2 and 3, respectively, felt certain (very or somewhat) about the appropriateness of their antibiotic use (Figure S1); only 7% (22/314) in all three hospitals felt very certain. More participants in Hospital 1 (97%) and Hospital 2 (94%) reported following treatment guidelines than Hospital 3 (78%), whereas more participants in Hospital 3 (90%) used antibiograms to inform treatment decisions than Hospital 1 (58%) and Hospital 2 (73%). Overall, 9% of all participants (29/317) reported they sometimes prescribed antibiotics even when not needed but in response to patients’ expectation, and 13% (40/316) prescribed to avoid complications. Many participants also believed that antibiotics were prescribed for longer than recommended (50%, 161/319) and broad-spectrum antibiotics were used when narrow-spectrum antibiotics were sufficient (49%, 156/319).

Overall, most participants agreed that AMR is a significant problem causing a large disease burden and that overuse of antibiotics can lead to AMR and waste of resources (Figure S2). About one-third to one-half of the participants were concerned that there was little interest among doctors in proper antibiotic use and AMR, and a lack of effective guiding policies in their hospitals. Most agreed on the likely impact of AMS interventions, except for participants in Hospital 3: 49% (68/138) disagreed and 39% (54/138) stayed neutral to the statement that infectious disease consultations can help ensure proper use of antibiotics (Figure S3). This was also consistent with the higher proportion of participants in Hospital 3 than in the other two hospitals who disagreed with the statement on having access to consultations from infectious disease doctors and clinical pharmacists (Figure S1).

Principal components analyses including all statements on AMR, AMS and prescribing practices identified six underlying components: perceptions about AMR in general, perceptions about suboptimal antibiotic use practices locally, perceptions about the impact of AMS programmes, support for prescribing practices, prescribing confidence and prescribing antibiotics when not needed. Table 2 describes the composition of statements for each identified component and the composite scores calculated based on the loadings of each statement to the components. The results of parallel analysis (Figure S4) were used to examine the components, and loadings of each statement to the six components are provided in Table S2. Participants had high mean composite scores for perceptions about AMR in general, and for perceptions about AMS impact. The levels of prescribing antibiotics when not needed were low across the three hospitals. These six components were used as variables in the multivariable analysis (see below) to identify associations with antibiotic prescribing practices.

**Table 2.** Six underlying components identified from the principal components analyses and the statements constituting each component

Component	Statements constituting component	Mean score (SD)^a^
1. Perceptions about AMR in general	Antimicrobial resistance is a significant countrywide problem A patient is likely to become infected with a multidrug-resistant pathogen during their stay at my hospital Overuse of antimicrobials can lead to antimicrobial resistance and waste of resources Infection with multidrug-resistant organisms is associated with increased mor­bidity (including length of stay) and mortality My individual effort at appropriate use of antibiotics can help with the hospital’s resistance problems	4.45 (0.53) 4.52 (0.54) 4.62 (0.57)
2. Perceptions about suboptimal antibiotic use locally	Antibiotics are overused in my hospital and in other hospitals of the country There is little interest among physicians at my hospital in the subject of proper use of antibiotics and control of antimicrobial resistance There is a lack of effective hospital policies to guide appropriate antimicrobial use Using antibiotics with longer than recommended duration occurs in this hospital Using broad-spectrum antibiotics when an antibiotic with narrower spectrum would be sufficient occurs in this hospital	3.38 (0.77) 3.08 (0.74) 3.37 (0.74)
3. Support for prescribing practices	I am aware of the antimicrobial resistance rates and patterns in my hospital Consultation from an infectious disease doctor can help ensure the proper use of antibiotics I have access to consultations from infectious diseases doctors in proper use of antimicrobials in this hospital I have access to consultations from clinical pharmacists in proper use of antimicrobials in this hospital	4.11 (0.42) 3.82 (0.53) 2.77 (0.43)
4. Perceptions about impact of AMS programme	Antimicrobial stewardship at this hospital is urgently needed The development of a hospital guideline on antimicrobial use based on local evidence would be more useful than following international guidelines Documenting the indication, dose and duration for all courses of antibiotics can help ensure that antibiotics are used in an appropriate manner Increasing education on antimicrobial use and resistance can help improve antibiotic prescribing in my hospital Requiring approval before the use of certain ‘restricted’ antibiotics is an effective way to reduce inappropriate antibiotic use Access to timely consultation from a clinical pharmacist on the proper dosing of antibiotics (e.g. when patients have renal insufficiency) is an effective way to reduce inappropriate antibiotic use	4.02 (0.57) 3.91 (0.74) 3.95 (0.66)
5. Prescribing confidence	How certain do you feel about the appropriateness of your antibiotic use? How often do you follow the recommendations of your hospital antimicrobial guidelines? I utilize the hospital antibiogram to help me to choose the proper antimicrobial treatment for patients	3.83 (0.84) 4.05 (0.80) 4.18 (0.76)
6. Prescribing antibiotics when not needed	I sometimes prescribe antibiotics for patients that don’t need them because I feel that the patient expects to receive antibiotics and will be dissatisfied if I do not prescribe them I sometimes prescribe antibiotics for patients that don’t need them because I want to avoid any potential complications in case the patient will not improve with other therapies	2.40 (0.73) 2.42 (0.86) 2.36 (0.78)

^a^Mean score (SD) for Hospital 1, Hospital 2 and Hospital 3, respectively, for each component, calculated using principal psych function with oblimin rotation, on a scale from 1 to 5 for strongly disagree to strongly agree or equivalent in response to the statements constituting each component.

### Variables associated with antibiotic prescribing practices

In the multivariable analysis (Table 3) with all variables added to the mixed effects model, higher antibiotic prescribing confidence was found among those with higher levels of perceptions about the impact of AMS programmes (score increased by 0.31 for prescribing confidence when score for AMS perception increased by 1, *P* < 0.001), and those in managerial positions (score for prescribing confidence was greater by 0.33 compared with those not in managerial positions, *P* = 0.05). Perceptions about suboptimal antibiotic use practices locally reduced confidence in antibiotic prescribing (reduced by 0.27 when score for perceptions about suboptimal antibiotic use practices locally increased by 1, *P* < 0.001). The level of prescribing antibiotics when not needed was negatively associated with the level of prescribing confidence (increased score of 1 in prescribing confidence associated with a reduction in the level of prescribing antibiotics when not needed by 0.17, *P* = 0.042). Prescribing antibiotics when not needed tended to occur more often among those who agreed (score increased by 0.44, *P* = 0.014) or stayed neutral (score increased by 0.57, *P* = 0.0024) with the statement on personal preference for branded antibiotics.

**Table 3.** Variables significantly associated with antibiotic prescribing confidence and prescribing when not needed in a multivariable model

Variable^a^	Prescribing confidence	Prescribing antibiotics when not needed
Age^b^	0.014 (0.16)	0.0027 (0.82)
Sex^c^ (reference: male)		
Female	0.061 (0.57)	0.21 (0.096)
Hospital type (reference: paediatric hospital)		
General hospital	0.031 (0.20)	−0.13 (0.64)
Specialty^c^ (reference: internal medicine)		
ICU	0.17 (0.21)	−0.048 (0.77)
Surgery	0.067 (0.68)	0.31 (0.11)
Infectious diseases	0.018 (0.91)	0.26 (0.16)
Others	0.025 (0.85)	−0.089 (0.58)
Experience in the current hospital^c^ (reference: <1 year)		
1–5 years	0.085 (0.61)	0.075 (0.70)
6–10 years	0.30 (0.12)	0.059 (0.79)
>10 years	0.060 (0.80)	0.25 (0.37)
Position in the current hospital^c^ (reference: managerial)		
Non-managerial	**−0.33** (**0.050)**	0.16 (0.42)
Training on antibiotics in past year^c^ (reference: No)		
Yes	0.090 (0.46)	−0.084 (0.56)
Perceived training on antibiotics was inadequate^c^ (reference: Agree)		
Disagree or neutral	0.10 (0.27)	−0.11 (0.33)
Preference for brand-name drugs^c,d^ (reference: Disagree)		
Neutral	−0.22 (0.15)	**0.44** (**0.014)**
Agree	0.18 (0.27)	**0.57** (**0.0024)**
Antibiotic prescribing frequency^c,e^ (reference: <20%)		
20%–39%	0.054 (0.79)	0.36 (0.13)
40%–59%	0.088 (0.60)	0.037 (0.85)
60%–79%	0.065 (0.71)	0.15 (0.47)
≥80%	0.084 (0.64)	0.23 (0.28)
Knowledge score^b^	0.016 (0.44)	−0.028 (0.26)
Perceptions about AMR in general^b^	0.13 (0.14)	−0.038 (0.72)
Perceptions about suboptimal practices locally^b^	**−0.27** (**0.000054)**	0.13 (0.12)
Perceptions about impact of AMS^b^	**0.31** (**0.000052)**	−0.065 (0.48)
Prescribing confidence^b^	Not applicable	**−0.17** (**0.042)**
Prescribing antibiotics when not needed	**−0.12** (**0.043)**	Not applicable

^a^Identified in a linear mixed model fit by restricted maximum likelihood using the lmer function in the lme4 package. All variables were added to the model as fixed effect, hospital ID added as random effect. Data presented for variables with *P* < 0.05 are highlighted in bold face.

^b^For continuous independent variables, data presented are the mean score increase in the outcome variable for every unit increase in the independent variable (*P* value in parentheses).

^c^For categorical independent variables, data presented are score difference between the mean of the outcome variable for the respective category compared with the reference category of independent variable (*P* value in parentheses).

^d^Response to statement: ‘I often prefer branded antibiotics over domestic/generic drugs in treatment of severe infections because I suspect that the latter are of poor quality and can affect the outcome of the patients.’

^e^Response to question: ‘Of the patients that you see on an average day, to what percentage do you usually prescribe antibiotics?’

## Discussion

This study has described self-reported antibiotic prescribing practices and related knowledge and perceptions in three tertiary hospitals in Vietnam, and identified factors that influenced practices of local doctors. Our study demonstrated a high level of uncertainty among doctors about their treatment appropriateness and the notable frequency of unnecessary antibiotic prescription (9% in response to patient expectation and 13% to avoid complications), and we have demonstrated that improving prescribing confidence can help to reduce unnecessary antibiotic prescribing. A few surveys in other countries consistently reported higher proportions of prescribing antibiotics following patient expectation; however, these data mostly were in primary and outpatient settings and for respiratory tract infections,^30,31^ but not in hospital settings, among the studies using KAP designs reviewed recently.^5^ Our estimate was obtained in tertiary settings with more severe infections requiring antibiotic treatment, and based on self-reported data.

Decisions to prescribe antibiotics when not needed to avoid complications can be due to clinical diagnostic uncertainty.^32,33^ In our previous survey among medical doctors in Vietnam, broad-spectrum antibiotics or combinations were more likely to be prescribed in the scenario of undifferentiated sepsis or in more severe infections.^8^ AMS programmes can support doctors to make more appropriate prescribing decisions and avoid overprescribing antibiotics when they are not necessary through enabling interventions and policies such as implementing biomarker point-of-care tests,^34^ promoting guideline compliance, and providing feedback to doctors on their prescribing practices.^35^ Our findings of the association between doctors’ preference for branded drugs and inappropriate prescribing warrants attention. A recent review reported a higher tendency to prescribe branded drugs among doctors in low-income countries than in other countries.^36^ Doctors’ perceptions on efficacy and safety of generic drugs can vary depending on the local healthcare system, drug policies, and availability of and access to drug information sources. Nonetheless, a systematic review in 2014 found no evidence of inferiority of generics compared with the innovators but was unable to confirm that they were equivalent due to lack of quality studies.^37^ AMS programmes should work to address doctors’ concerns related to drug quality in their local setting. Ensuring access to quality-assured, safe, effective and affordable antimicrobials remains an important element in AMS implementation at both national and facility level, as reflected in the most recent policy guidance by the WHO.^38^

Doctors’ perceptions about AMS impact on antibiotic use could increase prescribing confidence among doctors, as shown in our analysis. The AMS perceptions score represents a set of doctors’ views on various AMS interventions including hospital guideline based on local evidence, documentation of indication, dose and duration of antibiotics, education, preauthorization of restricted antibiotics and consultation from clinical pharmacy on antibiotic dosing. Doctors with positive views on the effects of these interventions were more certain about the appropriateness of their prescriptions and used antibiograms more often. This finding has not been reported elsewhere, and it indicates the importance of increasing the involvement of doctors in AMS-related activities in order to boost their confidence in their prescribing practices. However, prescribing confidence was negatively affected by doctors’ perception of their colleagues’ suboptimal practices, and was not associated with individual knowledge of antibiotic treatment. Previous studies have also shown a lack of correlation between knowledge and confidence in antibiotic prescribing practices.^39,40^ Confidence was among the important intrinsic factors influencing inappropriate antibiotic prescribing, and could be improved through implementation of rapid diagnostic tests, treatment policies or guidelines, or delayed prescribing to reduce diagnostic uncertainty.^33^ Regarding knowledge, our study showed an average level of knowledge with a mean score of 9.77 out of 16 (equivalent to 61.1%), which is similar to the score reported among 761 doctors in a cross-sectional survey in China (6.29 out of 10, equivalent to 62.9%).^41^ Improving knowledge remains essential in AMS programmes, and reviews have shown that educational interventions proved effective in improving antibiotic use in hospital and primary care settings.^42^

With the purposes of assessing the baseline status to inform AMS programmes, we designed the KAP survey to tailor specific components including knowledge and attitudes on antibiotic use and resistance, antibiotic decisions in common clinical case scenarios, and the specific AMS elements and actions recommended in the national guidelines. There have been a number of studies examining doctors’ attitudes and knowledge as factors affecting their antibiotic prescribing behaviour^5^; however, these have not been designed specifically to inform the development of AMS interventions at acute-care hospital settings, particularly in LMIC, as in our study. Through the knowledge questions, we identified the important and consistent gaps in knowledge across the three hospitals that need to be targeted in ongoing education and training of doctors. Although the results came from only three hospitals, the consistency in the knowledge gaps suggests common issues among Vietnamese doctors in antibiotic use practices in relation to antibiotic adjustment based on microbiology results, clinical pharmacology (antibiotic dosing to renal function, vancomycin monitoring), antibiotic choice in surgical prophylaxis, management of asymptomatic bacteriuria, community- and hospital-acquired pneumonia, and antibiotic anaphylaxis.

In addition, the low use of antibiograms to inform antibiotic treatments in two of the three hospitals points to the need to improve clinical microbiology in these hospitals. Programmes also need to address the proportion of doctors who still prescribe antibiotics in response to patients’ expectations or to avoid complications, as well as the need to reduce unnecessary antibiotic use through shortening antibiotic durations and increasing the use of narrow-spectrum antibiotics. Our KAP results also highlight the importance of having access to advice and consultations from infectious diseases experts and clinical pharmacists to support doctors in making appropriate antibiotic decisions. We have reported how AMS interventions were developed in the two provincial hospitals based on the baseline data including these KAP results that informed the content of the training activities and the increased support of clinical pharmacists through prospective audits and feedback to prescribers.^19^ At the national paediatric hospital, specific guidelines for antibiotic use have also been subsequently developed including those for surgical prophylaxis with initial promising outcomes reported.^43^

Besides the strengths of using principal components analysis as a method to reduce a large number of observed items to a smaller set of underlying components, this study also included both general and paediatric hospitals, with variations in hospital size and geographical location, and views of different clinical specialties. The study findings should be interpreted in light of its limitations. This was a self-administered survey; participants’ responses might have been biased during completion of the questionnaire through seeking the correct answers from other people or reference resources. Such potential bias might be small because the level of knowledge in our sample was similar to that of other studies in similar settings. Additionally, we added individual hospitals as a random effect in the multivariable model to address the cluster effect, where participants in the same hospital might share some similarity in their responses. The self-reported data collection method might have led to the low estimates of frequency of prescribing antibiotics when not needed. The second limitation comes from the non-probability, purposive and convenience sampling approach, although it is unclear how this might have affected the reported estimations.

In summary, doctors in our study had a moderate level of certainty about their antibiotic treatment decisions and positive perceptions about the impact of AMS programmes and interventions to improve antibiotic use in hospitals. Overprescribing practices were still common and should be the focus of AMS interventions at hospital settings in LMIC. Specific interventions should target the areas with opportunity for improvement in each hospital including improving knowledge through education and training, enhancing AMS support from a multidisciplinary team with infectious disease expertise, clinical pharmacy and microbiology, and increasing availability and utility of resources such as guidelines and policies for antibiotic use in each hospital. Our findings have important implications for designing contextualized AMS programmes to effectively address the specific needs and gaps in resource-limited settings like Vietnam.

## Supplementary Material

dlae064_Supplementary_Data

## Data Availability

Data may be obtained from the hospitals participating in this study when a data sharing agreement is in place.

## Supplementary data

Figures S1 to S4 and Tables S1 and S2 are available as Supplementary data at *JAC-AMR* Online.
