# Supplementary material for: Antibiotic prescribing practices of medical doctors in a resource-limited setting and the influence of individual perceptions and stewardship support: a survey in three tertiary hospitals in Vietnam
Source: JAC Antimicrob Resist. 2024 Apr 16;6(2):dlae064. doi: 10.1093/jacamr/dlae064 (PMC11022071; doi:10.1093/jacamr/dlae064)
Supplement: dlae064_Supplementary_Data [file dlae064_supplementary_data.docx]

**Supplementary Material**

**Method**

Survey form

**BACKGROUND INFORMATION**

1. Year of Birth:
2. Gender

Male Female

1. What is your highest academic degree?

Medical Doctor Specialist Level 1 Specialist Level 2

PhD Masters Others (please specify):

1. What is your primary work area in the hospital? (choose one)

Medicine (non-surgical) Intensive care unit (any type)

Pharmacy Surgery

Infectious Diseases Infection Control

Other (please specify):

1. How long have you worked in this institution?

Less than 1 year 1 to 5 years

6 to 10 years More than 10 years

1. What is your staff position in the hospital?

Department leader Staff physician

Resident Other (please specify):

1. How long have you worked in your current specialty or profession?

Less than 1 year 1 to 5 years

6 to 10 years More than 10 years

1. **Perception on Antimicrobial use and Antimicrobial Resistance**

| 1. Antimicrobial resistance is a significant countrywide problem | Strongly agree  Agree  Neutral  Disagree  Strongly disagree |
| --- | --- |
| 1. A patient is likely to become infected with a multidrug resistant pathogen during their stay at my hospital | Strongly agree  Agree  Neutral  Disagree  Strongly disagree |
| 1. I am aware of the antimicrobial resistance rates and patterns in my hospital | Strongly agree  Agree  Neutral  Disagree  Strongly disagree |
| 1. Antibiotics are overused in my hospital and in other hospitals of the country | Strongly agree  Agree  Neutral  Disagree  Strongly disagree |
| 1. Overuse of antimicrobials can lead to antimicrobial resistance and waste of resources. | Strongly agree  Agree  Neutral  Disagree  Strongly disagree |
| 1. Infection with multidrug resistant organisms (MDRO) is associated with increased mor­bidity (including length of stay) and mortality | Strongly agree  Agree  Neutral  Disagree  Strongly disagree |
| 1. There is little interest among physicians at my hospital in the subject of proper use of antibiotics and control of antimicrobial resistance. | Strongly agree  Agree  Neutral  Disagree  Strongly disagree |
| 1. There is a lack of effective hospital policies to guide appropriate antimicrobial use | Strongly agree  Agree  Neutral  Disagree  Strongly disagree |
| 1. My individual effort at appropriate use of antibiotics can help with the hospital’s resistance problems | Strongly agree  Agree  Neutral  Disagree  Strongly disagree |

1. **Education Needs on Antibiotics and Appropriate Use of Antibiotics**

| 1. During the last year, how many hours have you received education on antibiotics as part of training courses or facility activities? | 0 hrs  <4 hrs  4-7 hrs  8-12 hours  > 12 hours |
| --- | --- |
| 1. Do you agree or disagree with the following statement: The training on antimicrobial prescribing and use at the hospital is inadequate? | Strongly agree  Agree  Neutral  Disagree  Strongly disagree |
| 1. Please rank the following topics and activities of education on proper antimicrobial from your most needed (1) to least needed (5):   Proper use of Microbiology diagnostics  Pharmacological aspects of antibiotics  Antibiotic classes  De-escalating antibiotics  Case discussions of antibiotic challenges  Use of guidelines in clinical practice  More topics, please specify: |  |

1. **Prescription Practice**

| 1. Of the patients that you see on an average day, in what % do you usually prescribe antibiotics? | <20%  20 to 39%  40 to 59%  60 to 79%  ≥80% |
| --- | --- |
| 1. How certain do you feel about the appropriateness of your antibiotic use? | Very certain  Somewhat certain  Neither certain nor uncertain  Somewhat uncertain  Very uncertain |
| 1. How often do you follow the recommendations of your hospital antimicrobial guidelines? | Never  Rarely  Sometimes  Most of the time  Always  I am not aware of the hospital guidelines |
| 1. Please rank how often you use the following resources when prescribing antimicrobial therapy, from most used (1) to least used (6)   Guidelines of the hospital  Vietnam National guidelines  Up-to-date, Dynamed, or other on-line clinical support tools  Sanford guide, or other pocket guides  Internet search through Google or Bing  Printed textbooks |  |
| 1. I utilize the hospital antibiogram to help me to choose the proper antimicrobial treatment for patients. | Never  Rarely  Sometimes  Most of the time  Always  I am not aware of an antibiogram being available at my hospital |
| 1. I have access to consultations from Infectious Diseases Doctors in proper use of antimicrobials in this hospital. | Strongly agree  Agree  Neutral  Disagree  Strongly disagree |
| 1. I have access to consultations from Clinical Pharmacists in proper use of antimicrobials in this hospital. | Strongly agree  Agree  Neutral  Disagree  Strongly disagree |
| 1. I often prefer branded antibiotics over domestic / generic drugs in treatment of severe infections because I suspect that the later are of poor quality and can affect the outcome of the patients | Strongly agree  Agree  Neutral  Disagree  Strongly disagree |
| 1. I sometimes prescribe antibiotics for patients that don’t need them because I feel that the patient expects to receive antibiotics and will be dissatisfied if I do not prescribe them. | Strongly agree  Agree  Neutral  Disagree  Strongly disagree |
| 1. I sometimes prescribe antibiotics for patients that don’t need them because I want to avoid any potential complications in case the patient will not improve with other therapies. | Strongly agree  Agree  Neutral  Disagree  Strongly disagree |
| 1. Using antibiotics with longer than recommended duration occurs in this hospital. | Strongly agree  Agree  Neutral  Disagree  Strongly disagree |
| 1. Using broad-spectrum antibiotics when an antibiotic with narrower spectrum would be sufficient occurs in this hospital. | Strongly agree  Agree  Neutral  Disagree  Strongly disagree |
| 1. The hospital’s policy on restricted antibiotics is strictly followed at your clinical department | Strongly agree  Agree  Neutral  Disagree  Strongly disagree |

1. **Effectiveness of Antimicrobial Stewardship Program**

| 1. Antimicrobial Stewardship at this hospital is urgently needed | Strongly agree  Agree  Neutral  Disagree  Strongly disagree  Don’t know about Antimicrobial Stewarship program |
| --- | --- |
| 1. The development of a hospital guideline on antimicrobial use based on local evidence would be more useful than following international guidelines | Strongly agree  Agree  Neutral  Disagree  Strongly disagree |
| 1. Documenting the indication, dose, and duration for all courses of antibiotics can help ensure that antibiotics are used in appropriate manner | Strongly agree  Agree  Neutral  Disagree  Strongly disagree |
| 1. Consultation from an Infectious Disease doctor can help ensure the proper use of antibiotics | Strongly agree  Agree  Neutral  Disagree  Strongly disagree |
| 1. Increasing education on antimicrobial use and resistance can help improve antibiotic prescribing in my hospital | Strongly agree  Agree  Neutral  Disagree  Strongly disagree |
| 1. Requiring approval before the use of certain ‘restricted’ antibiotics is an effective way to reduce inappropriate antibiotic use | Strongly agree  Agree  Neutral  Disagree  Strongly disagree |
| 1. Access to timely consultation from a clinical pharmacist on the proper dosing of antibiotics (e.g. when patients have renal insufficiency) is an effective way to reduce inappropriate antibiotic use. | Strongly agree  Agree  Neutral  Disagree  Strongly disagree |

1. **Knowledge on Antibiotics and Antimicrobial resistance**

| 1. All of the following are considered best practice in taking blood cultures, EXCEPT 2. Taking the sample when a patient has signs and symptoms of infection 3. Taking at least 2 samples from different sites 4. Taking the sample if the patient does not improve after the first dose of antibiotics 5. Disinfect the venipuncture site prior to taking the sample. | A  B  C  D  Don’t know |
| --- | --- |
| 1. Which one of the following antibiotics has the best activity against anaerobes? | Ciprofloxacin  Metronidazole  Trimethoprim-sulfamethoxazole  Vancomycin  Don’t know |
| 1. Which one of the following antibiotic is most effective in crossing the blood-brain barrier? | Clindamycin  Ceftriaxone  Vancomycin  Azithromycin  Don’t know |
| 1. A patient was brought to the clinic complaining of fever (39 ^o^C), malaise, and nasal discharge for 3 days. Which treatment would you recommend? | Amoxicillin  Trimethoprim-sulfamethoxazole  Azithromycin  No antibiotic, give symptomatic treatment  Don’t know |
| 1. A patient was admitted to the hospital because of severe pneumonia with moderate respiratory failure. The chest X-ray showed infiltrate in the right lower lobe and moderate pleural effusion. The patient was started on ampicillin/clavulanate and vancomycin and she improved after 3 days. The blood culture result came back positive for *Streptococcus pneumoniae*; sensitive to penicillin. Which antibiotic would you use to continue treatment for the patient? | Vancomycin  Ampicillin  Ceftriaxone  Continue Ampicillin/Clavulanate plus Vancomycin  Don’t know |
| 1. A patient was admitted because of fever and a pyomyositis after an open trauma wound. The blood culture grew *Staphylococcus aureus* resistant to oxacillin. What is the recommended antibiotic therapy? | Methicillin  Ceftriaxone  Vancomycin  Meropenem  Don’t know |
| 1. Which of the following antibiotics does not require dose adjustment in case of impaired kidney function? | Clindamycin  Gentamicin  Ceftazidime  Vancomycin  Don’t know |
| 1. A patient undergoing evaluation for some endocrine abnormalities was found to have pyuria with a white blood cell count of 300 cells/mm3 and the urine culture grew *Escherichia coli* of 10^3^/ml. She does not have either fever, dysuria or tenderness over the suprapubic area. Which treatment is appropriate for this girl? | No antibiotics needed  Cephazolin  Ciprofloxacin  Trimethoprime-sulphamethoxazole  Don’t know |
| 1. A patient is diagnosed with appendicitis and about to have appendectomy. Which antibiotic(s) should she have as surgical prophylaxis? | No antibiotics needed  Cefuroxime  Ciprofloxacin and vancomycin  Trimethoprime-sulphamethoxazole  Don’t know |
| 1. You are taking care of a patient with ampicillin resistant enterococcal endocarditis. The patient is receiving vancomycin twice a day. Which of the following level determinations is most often recommended to monitor vancomycin therapy lasting several days? | Vancomycin “peak” concentration  Vancomycin “trough” concentration  Vancomycin levels are not required  Don’t know |
| 1. A patient is being treated for his *Klebsiella* septicemia with ceftazidime and amikacin. The patient has moderate level of renal insufficiency associated with sepsis. Which strategy for amikacin dosing would be most effective in a patient with impaired renal function? 2. Decrease the dose and extend the infusion duration from 30 minutes to 3 hours. 3. Administer the current dose but extend the dos­ing interval from every 8 to every 12 hours or longer in accordance with the level of renal function impairment 4. Discontinue the drug | A  B  C  Don’t know |
| 1. A patient was seen in the Outpatient department with a subcutaneous abscess of 3 cm in diameter in the buttock with erythema and swelling 7 cm around the abscess. Which of the following would be the best management approach? 2. Drain the abscess and give oral cephalexin 3. Drain the abscess and give oral ciprofloxacin 4. Drain the abscess and give cephazolin IV and gentamicin 5. Drain the abscess and give ceftazidime | A  B  C  D  Don’t know |
| 1. Switching from parenteral to oral antibiotics is preferred when possible. Which of the following conditions is NOT compatible for switching the antimicrobial drugs from parenteral use to oral? 2. High bioavailability of the drug when taken orally 3. The patient is tolerating oral feeding and can take other oral medications. 4. The patient’s condition has improved with the initial intravenous therapy 5. The patient has a malabsorption syndrome | A  B  C  D  Don’t know |
| 1. Which of the following is a recommended empiric therapy for a hospitalized patient with community-acquired pneumonia? | No antibiotics needed  Ceftriaxone  Meropenem  Ceftazidine  Don’t know |
| 1. An patient presents to the clinic with a right leg cellulitis. He reports anaphylaxis to penicillin 2 years ago. Which of the following would be an appropriate antibiotic choice? | Oxacillin  Cefazolin  Clindamycin  Gentamicin  Doxycyline  Don’t know |
| 1. A patient develops a new severe pneumonia 10 days after being admitted to the hospital with multiple injuries following a motorbike accident. While awaiting sputum culture results, which of the following would be an appropriate antibiotic choice? | Clindamycin  Ceftriaxone plus Azithromycin  Levofloxacin + Ceftazidime  Piperacillin-Tazobactam  Don’t know |

**Reference documents which were based on during the development of the survey form:**

- Viet Nam Ministry of Health. Quyết định về việc ban hành tài liệu "Hướng dẫn thực hiện quản lý sử dụng kháng sinh trong bệnh viện" [Decision on the issuance of the document "Guideline on implementing antimicrobial stewardship in hospitals"] (No. 772/QĐ-BYT). Hanoi: Viet Nam Ministry of Health; 2016.
- Viet Nam Ministry of Health. Hướng dẫn sử dụng kháng sinh [Antibiotic use guideline]. Hanoi: Medical Publishing House, 2015.
- Centers for Disease Control and Prevention (CDC). Acute Care Facility Multidrug-resistant Organisms Control Activity Assessment Tool. Accessed 26 Feb 2023; <https://www.cdc.gov/hai/pdfs/prevent/mdro-facility-assessment_7_28.pdf>
- Pollack LA, Srinivasan A. Core elements of hospital antibiotic stewardship programs from the Centers for Disease Control and Prevention. Clin Infect Dis. 2014;59 Suppl 3(Suppl 3):S97-100.
- Strengthening Pharmaceutical Systems. Infection control assessment tool. 2nd ed. Arlington, VA: Management Sciences for Health; 2009.
- Huskins C, Ross-Degnan D, Goldmann DA. Improving infection control in developing countries: the infection control assessment tool. BMC Proc. 2011 Jun 29;5(Suppl 6):O18. doi: 10.1186/1753-6561-5-S6-O18. PMCID: PMC3239428.
- Dellit TH, Owens RC, McGowan JE, Gerding DN, Weinstein RA, Burke JP, et al. Infectious Diseases Society of America and the Society for Healthcare Epidemiology of America Guidelines for Developing an Institutional Program to Enhance Antimicrobial Stewardship. Clin Infect Dis. 2007;44(2):159-77.

**Supplementary Tables:**

**Table S1.** Proportion of participants with correct answer to knowledge questions on antibiotic treatment. The number of doctors who responded to each of the question was variable across all questions, particularly in Hospital 2 with a high proportions of missing data for some questions.

| # | Question | Correct answer | Hospital 1 (n=61) | Hospital 2 (n=120) | Hospital 3  (n= 141) |
| --- | --- | --- | --- | --- | --- |
| q1 | All of the following are considered best practice in taking blood cultures, EXCEPT  Taking the sample when a patient has signs and symptoms of infection  Taking at least 2 samples from different sites  Taking the sample if the patient does not improve after the first dose of antibiotics  Disinfect the venipuncture site prior to taking the sample. | Taking the sample if the patient does not improve after the first dose of antibiotics | 43/61 (70.5%) | 86/120 (71.7%) | 102/132  (77.3%) |
| q2 | Which one of the following antibiotics has the best activity against anaerobes? | Metronidazole | 55/59 (93.2%) | 116/117 (99.1%) | 122/132 (92.4%) |
| q3 | Which one of the following antibiotic is most effective in crossing the blood-brain barrier? | Ceftriaxone | 52/60 (86.7%) | 94/117 (80.3%) | 118/132 (89.4%) |
| q4 | A patient was brought to the clinic complaining of fever (39 ^o^C), malaise, and nasal discharge for 3 days. Which treatment would you recommend? | No antibiotic, give symptomatic treatment | 41/61 (67.2%) | 50/62 (80.6%) | 118/132 (89.4%) |
| q5 | A patient was admitted to the hospital because of severe pneumonia with moderate respiratory failure. The chest X-ray showed infiltrate in the right lower lobe and moderate pleural effusion. The patient was started on ampicillin/clavulanate and vancomycin and she improved after 3 days. The blood culture result came back positive for *Streptococcus pneumoniae*; sensitive to penicillin. Which antibiotic would you use to continue treatment for the patient? | Ampicillin | 19/60 (31.7%)  (32/60 chose to continue ampicillin/ clavulanate plus vancomycin) | 5/62 (8.1%)  (54/62 chose to continue ampicillin/ clavulanate plus vancomycin) | 72/132 (54.5%)  (54/132 chose to continue ampicillin/ clavulanate plus vancomycin) |
| q6 | A patient was admitted because of fever and a pyomyositis after an open trauma wound. The blood culture grew *Staphylococcus aureus* resistant to oxacillin. What is the recommended antibiotic therapy? | Vancomycin | 45/61 (73.8%) | 47/54 (87.0%) | 125/132 (94.7%) |
| q7 | Which of the following antibiotics does not require dose adjustment in case of impaired kidney function? | Clindamycin | 27/60 (45.0%)  23/60 chose ceftazidim | 79/119 (66.4%)  14/119 chose ceftazidim | 47/132 (35.6%)  70/132 chose ceftazidim |
| q8 | A patient undergoing evaluation for some endocrine abnormalities was found to have pyuria with a white blood cell count of 300 cells/mm3 and the urine culture grew *Escherichia coli* of 10^3^/ml. She does not have either fever, dysuria or tenderness over the suprapubic area. Which treatment is appropriate for this girl? | No antibiotics needed | 14/60 (23.3%)  38/60 chose ciprofloxacin | 25/61 (41.0%)  24/61 chose ciprofloxacin | 72/132 (54.5%)  42/132 chose trimethoprim-sulphamethoxazol |
| q9 | A patient is diagnosed with appendicitis and about to have appendectomy. Which antibiotic(s) should she have as surgical prophylaxis? | Cefuroxime | 28/61 (45.9%) | 67/120 (55.8%) | 82/132 (62.1%) |
| q10 | You are taking care of a patient with ampicillin resistant enterococcal endocarditis. The patient is receiving vancomycin twice a day. Which of the following level determinations is most often recommended to monitor vancomycin therapy lasting several days? | Vancomycin “trough” concentration | 14/57 (24.6%)  31/57 chose vancomycin “peak” concentration | 23/120 (19.2%)  67/120 chose vancomycin “peak” concentration | 75/132 (56.8%)  43/132 chose vancomycin “peak” concentration |
| q11 | A patient is being treated for his *Klebsiella* septicemia with ceftazidime and amikacin. The patient has moderate level of renal insufficiency associated with sepsis. Which strategy for amikacin dosing would be most effective in a patient with impaired renal function?  - Decrease the dose and extend the infusion duration from 30 minutes to 3 hours.  - Administer the current dose but extend the dos­ing interval from every 8 to every 12 hours or longer in accordance with the level of renal function impairment  - Discontinue the drug | Administer the current dose but extend the dos­ing interval from every 8 to every 12 hours or longer in accordance with the level of renal function impairment | 21/61 (34.4%)  24/61 chose to decrease the dose and extend the infusion duration from 30 minutes to 3 hours. | 45/61 (73.8%)  15/61 chose to decrease the dose and extend the infusion duration from 30 minutes to 3 hours. | 55/132 (41.7%)  43/132 chose to decrease the dose and extend the infusion duration from 30 minutes to 3 hours. |
| q12 | A patient was seen in the Outpatient department with a subcutaneous abscess of 3 cm in diameter in the buttock with erythema and swelling 7 cm around the abscess. Which of the following would be the best management approach?  Drain the abscess and give oral cephalexin  Drain the abscess and give oral ciprofloxacin  Drain the abscess and give cephazolin IV and gentamicin  Drain the abscess and give ceftazidime | Drain the abscess and give oral cephalexin | 15/61 (24.6%)  18/61 chose to drain the abscess and give ceftazidime, 16/61 chose to drain the abscess and give cephazolin IV and gentamicin | 47/120 (39.2%)  34/120 chose to drain the abscess and give cephazolin IV and gentamicin | 81/132 (61.4%)  24/132 chose to drain the abscess and give cephazolin IV and gentamicin |
| q13 | Switching from parenteral to oral antibiotics is preferred when possible. Which of the following conditions is NOT compatible for switching the antimicrobial drugs from parenteral use to oral?  High bioavailability of the drug when taken orally  The patient is tolerating oral feeding and can take other oral medications.  The patient’s condition has improved with the initial intravenous therapy  The patient has a malabsorption syndrome | The patient has a malabsorption syndrome | 35/61 (57.4%) | 85/120 (70.8%) | 114/132 (86.4%) |
| q14 | Which of the following is a recommended empiric therapy for a hospitalized patient with community-acquired pneumonia? | Ceftriaxone | 36/61 (59.0%) | 24/120 (20.0%)  85/120 (70.8%) chose ceftazidime | 87/132 (65.9%) |
| q15 | A patient presents to the clinic with a right leg cellulitis. He reports anaphylaxis to penicillin 2 years ago. Which of the following would be an appropriate antibiotic choice? | Clindamycin | 29/60 (48.3%)  10/60 chose gentamicin | 21/62 (33.9%) 16/62 chose gentamicin | 67/132 (50.8%)  30/132 chose oxacillin |
| q16 | A patient develops a new severe pneumonia 10 days after being admitted to the hospital with multiple injuries following a motorbike accident. While awaiting sputum culture results, which of the following would be an appropriate antibiotic choice? | Levofloxacin + Ceftazidime | 39/59 (66.1%)  16/59 chose pipercillin-tazobactam | 71/120 (59.2%)  32/120 chose pipercillin-tazobactam | 46/132 (34.8%)  55/132 chose ceftriaxone + azithromycin |

**Table S2.** Results of principal component analysis for all items on AMR, AMS and prescribing practices in a six-factor solution

|  | Statement | Loadings for six-component solution* | | | | | |
| --- | --- | --- | --- | --- | --- | --- | --- |
|  |  | C1-4 | C2-2 | C3-3 | C4-1 | C5-5 | C6-6 |
| amr1 | Antimicrobial resistance is a significant countrywide problem | **0.74** | -0.16 | -0.035 | -0.059 | -0.065 | -0.039 |
| amr2 | A patient is likely to become infected with a multidrug resistant pathogen during their stay at my hospital | **0.85** | -0.059 | 0.060 | -0.11 | -0.065 | -0.039 |
| amr5 | Overuse of antimicrobials can lead to antimicrobial resistance and waste of resources | **0.67** | 0.14 | -0.0038 | 0.032 | 0.032 | -0.11 |
| amr6 | Infection with multidrug resistant organisms (MDRO) is associated with increased mor­bidity (including length of stay) and mortality | **0.77** | -0.014 | -0.10 | 0.016 | 0.027 | -0.15 |
| amr9 | My individual effort at appropriate use of antibiotics can help with the hospital’s resistance problems | **0.36** | -0.039 | -0.15 | 0.23 | 0.31 | 0.084 |
| amr4 | Antibiotics are overused in my hospital and in other hospitals of the country | 0.49 | **0.51** | 0.082 | -0.15 | -0.14 | 0.031 |
| amr7 | There is little interest among physicians at my hospital in the subject of proper use of antibiotics and control of antimicrobial resistance | -0.017 | **0.74** | 0.087 | 0.14 | -0.031 | -0.0090 |
| amr8 | There is a lack of effective hospital policies to guide appropriate antimicrobial use | 0.026 | **0.64** | 0.12 | -0.096 | -0.26 | 0.0052 |
| pp8 | Using antibiotics with longer than recommended duration occurs in this hospital. | -0.17 | **0.78** | -0.12 | 0.086 | 0.17 | -0.036 |
| pp9 | Using broad-spectrum antibiotics when an antibiotic with narrower spectrum would be sufficient occurs in this hospital. | -0.075 | **0.76** | -0.17 | 0.039 | 0.21 | 0.11 |
| amr3 | I am aware of the antimicrobial resistance rates and patterns in my hospital | 0.31 | 0.0027 | **0.45** | -0.036 | 0.20 | 0.031 |
| ams4 | Consultation from an Infectious Disease doctor can help ensure the proper use of antibiotics | 0.017 | 0.085 | **0.80** | -0.028 | -0.12 | -0.061 |
| pp4 | I have access to consultations from Infectious Diseases Doctors in proper use of antimicrobials in this hospital. | -0.00066 | 0.020 | **0.78** | 0.041 | 0.065 | 0.094 |
| pp5 | I have access to consultations from Clinical Pharmacists in proper use of antimicrobials in this hospital. | -0.049 | -0.11 | **0.84** | 0.12 | -0.077 | 0.0038 |
| ams1 | Antimicrobial Stewardship at this hospital is urgently needed | 0.21 | 0.16 | -0.041 | **0.51** | 0.11 | -0.14 |
| ams2 | The development of a hospital guideline on antimicrobial use based on local evidence would be more useful than following international guidelines | 0.069 | 0.046 | -0.047 | **0.61** | -0.076 | 0.010 |
| ams3 | Documenting the indication, dose, and duration for all courses of antibiotics can help ensure that antibiotics are used in appropriate manner | -0.078 | -0.040 | 0.18 | **0.77** | -0.015 | 0.092 |
| ams5 | Increasing education on antimicrobial use and resistance can help improve antibiotic prescribing in my hospital | 0.049 | -0.043 | 0.12 | **0.59** | -0.20 | -0.0051 |
| ams6 | Requiring approval before the use of certain ‘restricted’ antibiotics is an effective way to reduce inappropriate antibiotic use | 0.034 | 0.19 | -0.053 | **0.63** | 0.85 | -0.060 |
| ams7 | Access to timely consultation from a clinical pharmacist on the proper dosing of antibiotics (e.g. when patients have renal insufficiency) is an effective way to reduce inappropriate antibiotic use. | -0.16 | -0.023 | 0.014 | **0.84** | -0.13 | 0.0059 |
| pp1 | How certain do you feel about the appropriateness of your antibiotic use? | -0.047 | 0.10 | 0.22 | -0.13 | **0.71** | -0.13 |
| pp2 | How often do you follow the recommendations of your hospital antimicrobial guidelines? | -0.17 | -0.063 | 0.56 | 0.0023 | **0.53** | -0.0031 |
| pp3 | I utilize the hospital antibiogram to help me to choose the proper antimicrobial treatment for patients. | -0.012 | 0.044 | -0.23 | -0.17 | **0.80** | 0.15 |
| pp6 | I sometimes prescribe antibiotics for patients that don’t need them because I feel that the patient expects to receive antibiotics and will be dissatisfied if I do not prescribe them. | 0.0092 | 0.028 | 0.024 | 0.016 | 0.039 | **0.91** |
| pp7 | I sometimes prescribe antibiotics for patients that don’t need them because I want to avoid any potential complications in case the patient will not improve with other therapies. | -0.0062 | 0.057 | 0.012 | 0.034 | 0.038 | **0.90** |
| pp10 | The hospital’s policy on restricted antibiotics is strictly followed at your clinical department | 0.14 | -0.31 | -0.062 | 0.26 | 0.23 | -0.016 |

*Notes:*

*amr*: statements initially listed under antimicrobial resistance section in the questionnaire; *ams*: statements initially listed under antimicrobial stewardship section in the questionnaire; *pp*: statements initially listed under prescribing practices section in the questionnaire;

*using principal {psych} function, with Promax rotation, six components were extracted with the loadings of each statement to each component are presented in this stable; statement *pp10* did not contribute significantly to any of the component following the analysis.

Six extracted components: C1: perceptions about AMR in general, C2: perceptions about suboptimal antibiotic use practices locally, C3: support for prescribing practices, C4: perceptions about impact of AMS program, C5: prescribing confidence, and C6: prescribing antibiotics when not needed.

Description of mean composite scores across hospitals for the identified components:

Participants had high mean composite scores for perceptions about AMR in general (component 1) (4.45, 4.52 and 4.62, on the scale from 1 to 5: strongly disagree to strongly agree), and for perceptions about AMS impact (component 4) (4.02, 3.91 and 3.95 for Hospital 1, 2 and 3 respectively). The scores for perceptions about suboptimal antibiotic use practices locally were 3.38, 3.08, and 3.37 for Hospital 1, 2 and 3 respectively (component 2). Compared to Hospital 3 (mean score: 2.77), more support for prescribing practices were observed in Hospital 1 (4.11) and 2 (3.82) (component 3). However, prescribing confidence appears to be highest for Hospital 3 (mean score: 4.18), followed by Hospital 2 (4.05) and 1 (3.83) (component 5). The levels of prescribing antibiotics when not needed were low across three hospitals (mean score: 2.40, 2.42 and 2.36 for Hospital 1, 2 and 3 respectively) (component 6).


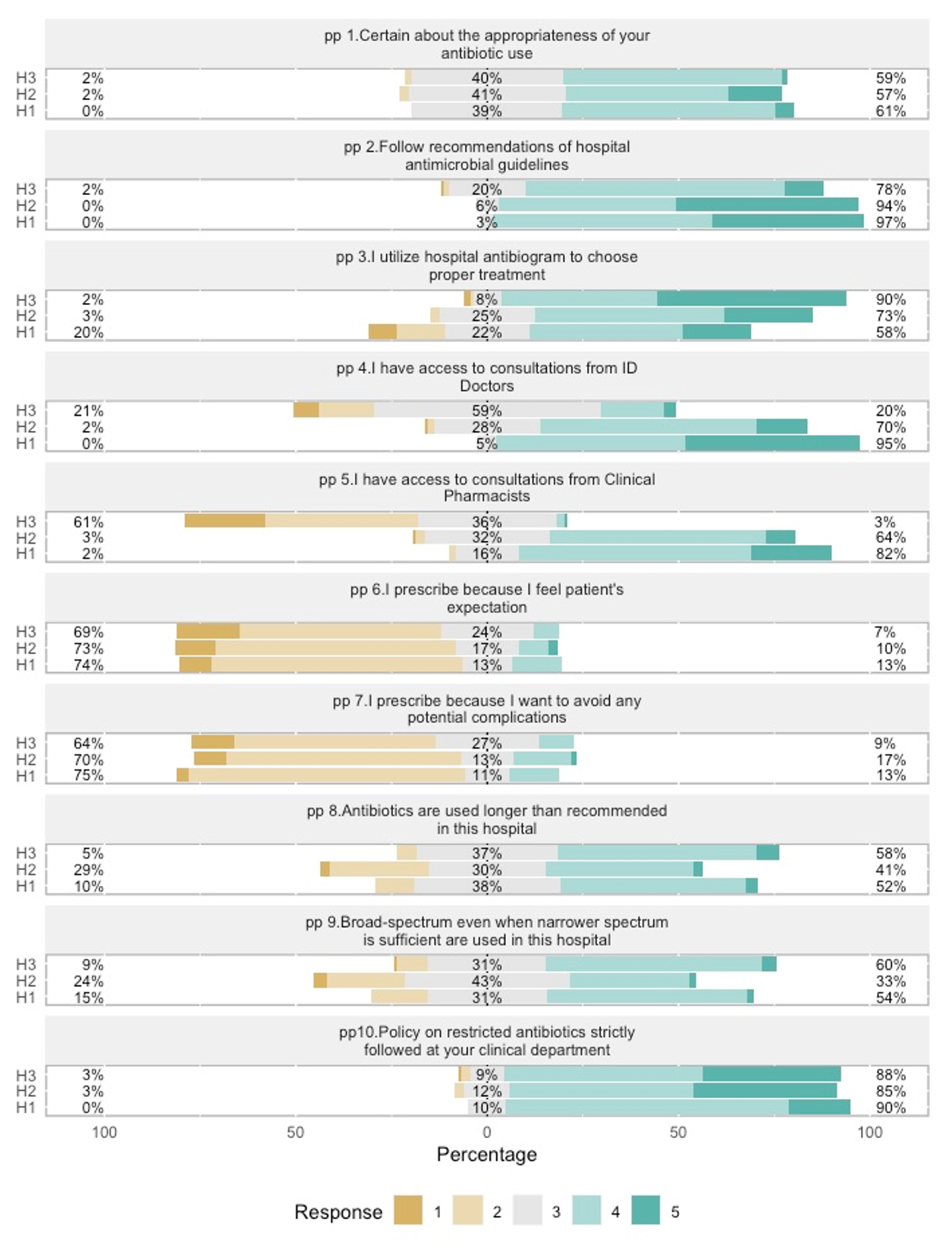


**Figure S1.** Percentage of participants responding to each statement on antibiotic prescribing practices (pp) by hospital; Note: Hospital 1: H1; Hospital 2: H2; Hospital 3: H3; Responses: 1=Strongly disagree, 2=Disagree, 3=Neutral, 4=Agree, 5=Strongly agree (except for pp1: 1=Very uncertain, 2=Somewhat uncertain, 3=Neither certain nor uncertain, 4=Somewhat certain, 5=Very certain, and or pp2: 1=Never, 2=Rarely, 3=Sometimes, 4=Most of the time, 5=Always); ID: infectious disease. The x-axis shows the percent scale from 0 to 100% to the left to indicate the responses 1 and 2, and to the right to indicate percent for the responses 4 and 5. The percent of response 3 is shown in the middle (centered around the 0%). The results for each statement is shown in each panel. For each statement, there are three bars in the panel that are depicted from low to high to represent the results of the responses for Hospital 1, Hospital 2 and Hospital 3, respectively. The area of the color shows the percent corresponding to each response category identified by the color legend from 1 to 5. For the data labels, for simplicity, we show the combined percentage value of response 1 and 2 on the left, the combined percentage value of response 4 and 5 on the right, and the percentage value of response 3 in the middle of the plot.


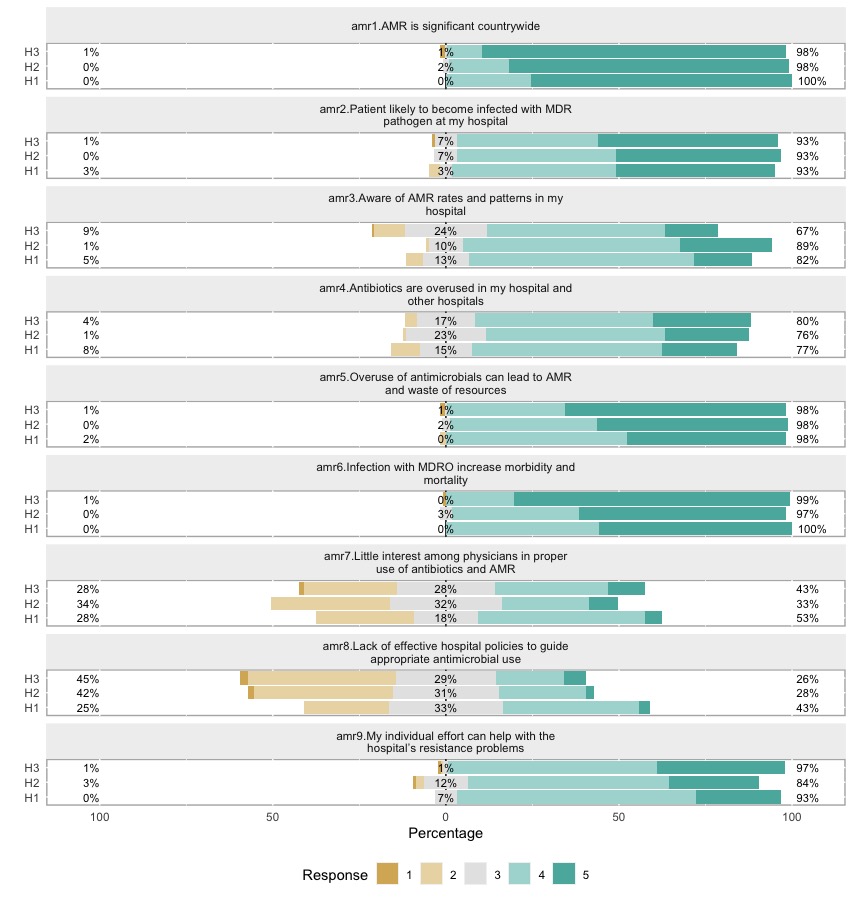


**Figure S2**. Percentage of participants responding to each statement on antimicrobial resistance (AMR) by hospital; Note: Hospital 1: H1; Hospital 2: H2; Hospital 3: H3; Responses: 1=Strongly disagree, 2=Disagree, 3=Neutral, 4=Agree, 5=Strongly agree; MDR: multidrug resistant; MDRO: multidrug resistant organism. The x-axis shows the percent scale from 0 to 100% to the left to indicate the responses 1 and 2, and to the right to indicate percent for the responses 4 and 5. The percent of response 3 is shown in the middle (centered around the 0%). The results for each statement is shown in each panel. For each statement, there are three bars in the panel that are depicted from low to high to represent the results of the responses for Hospital 1, Hospital 2 and Hospital 3, respectively. The area of the color shows the percent corresponding to each response category identified by the color legend from 1 to 5. For the data labels, for simplicity, we show the combined percentage value of response 1 and 2 on the left, the combined percentage value of response 4 and 5 on the right, and the percentage value of response 3 in the middle of the plot.


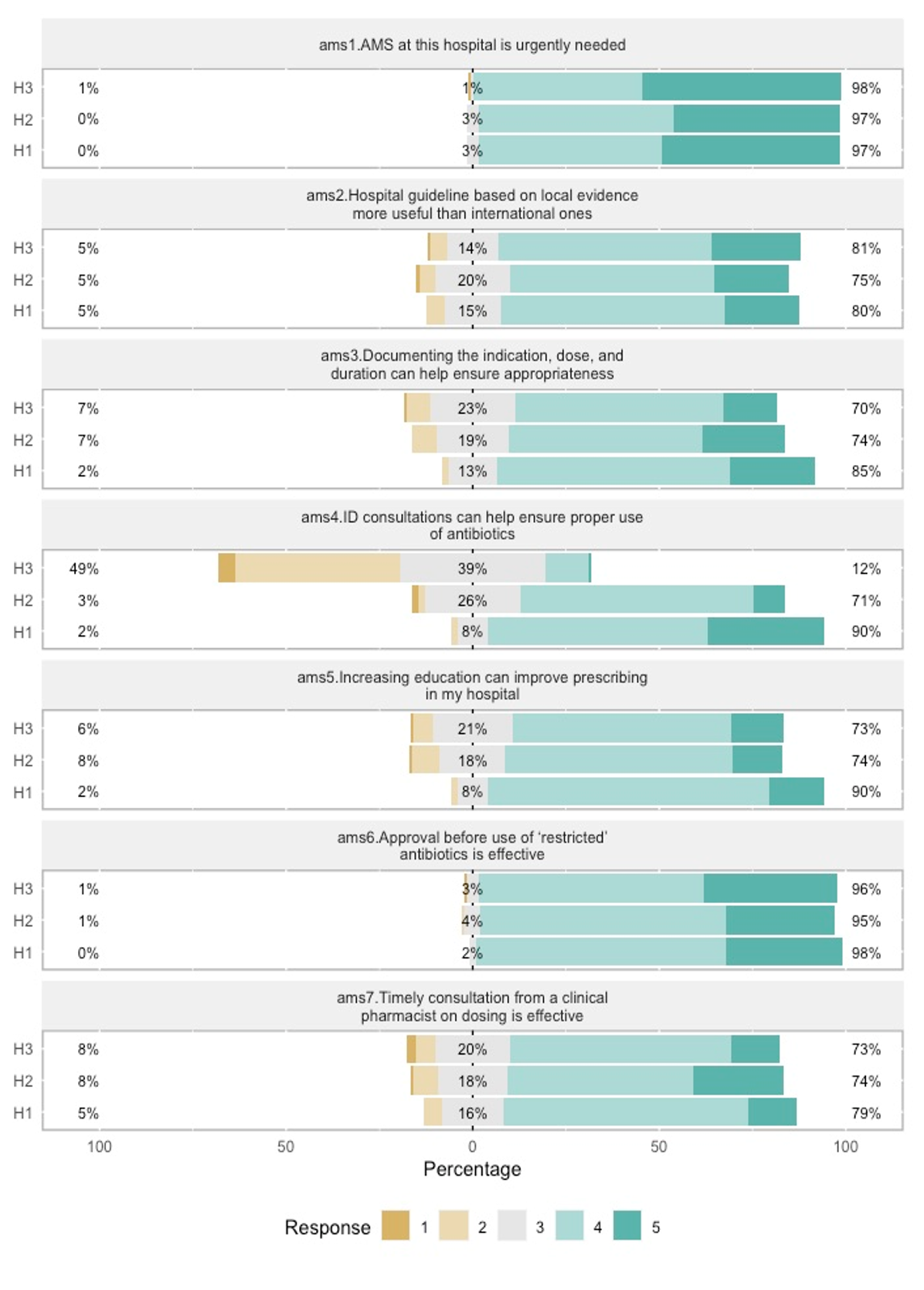


**Figure S3.** Percentage of participants responding to each statement on antimicrobial stewardship (AMS) by hospital; Note: Hospital 1: H1; Hospital 2: H2; Hospital 3: H3; Responses: 1=Strongly disagree, 2=Disagree, 3=Neutral, 4=Agree, 5=Strongly agree; ID: infectious disease. The x-axis shows the percent scale from 0 to 100% to the left to indicate the responses 1 and 2, and to the right to indicate percent for the responses 4 and 5. The percent of response 3 is shown in the middle (centered around the 0%). The results for each statement is shown in each panel. For each statement, there are three bars in the panel that are depicted from low to high to represent the results of the responses for Hospital 1, Hospital 2 and Hospital 3, respectively. The area of the color shows the percent corresponding to each response category identified by the color legend from 1 to 5. For the data labels, for simplicity, we show the combined percentage value of response 1 and 2 on the left, the combined percentage value of response 4 and 5 on the right, and the percentage value of response 3 in the middle of the plot.


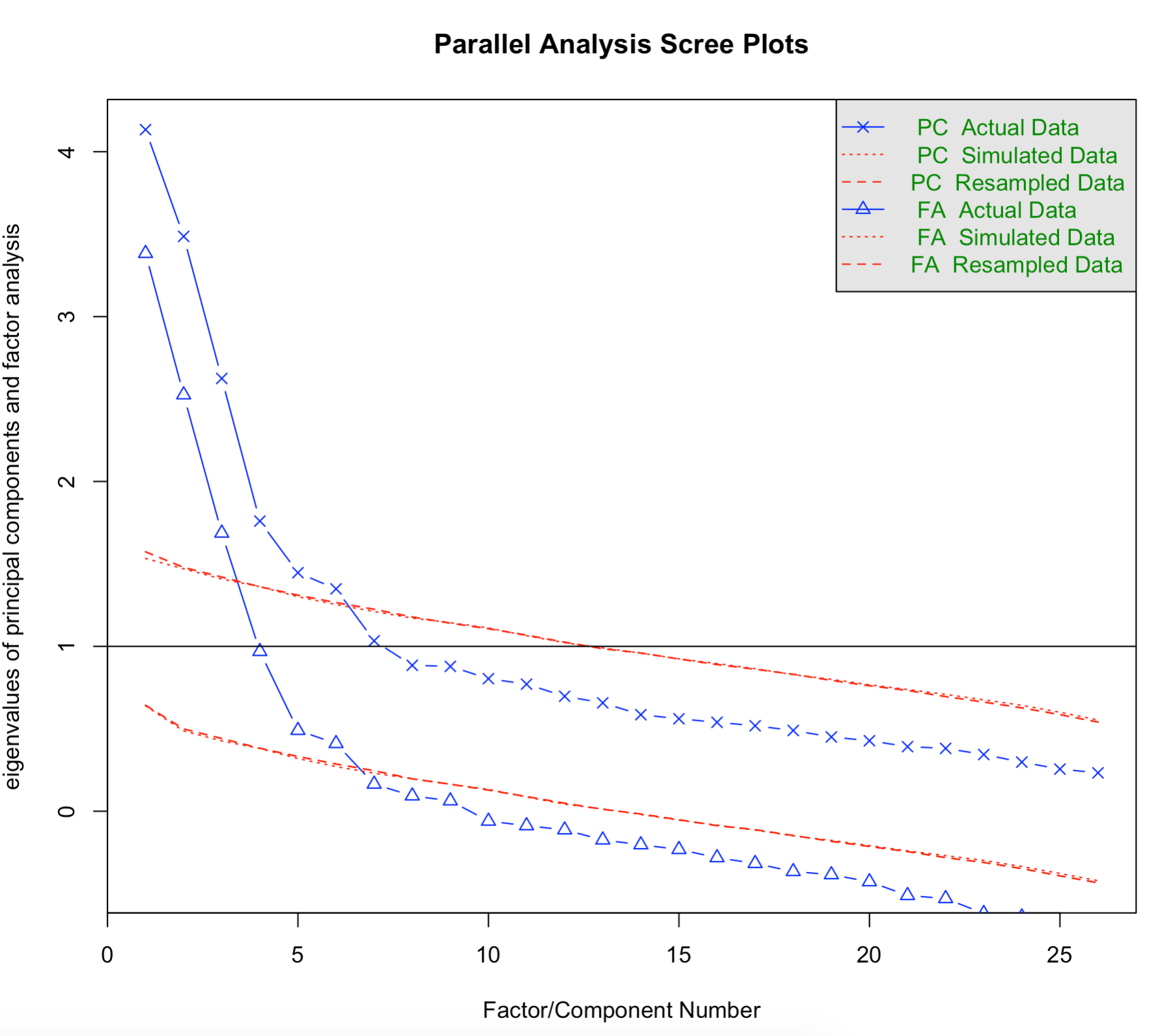


**Figure S4.** Parallel analysis suggesting that the number of components is 6 under the principal component (PC) analysis method, similar to the number of factors suggested in the factor analysis (FA) method.
